# Silibinin efficacy in a rat model of pulmonary arterial hypertension using monocrotaline and chronic hypoxia

**DOI:** 10.1186/s12931-019-1041-y

**Published:** 2019-04-25

**Authors:** Tingting Zhang, Nanako Kawaguchi, Kenji Yoshihara, Emiko Hayama, Yoshiyuki Furutani, Kayoko Kawaguchi, Takeshi Tanaka, Toshio Nakanishi

**Affiliations:** 1 Department of Pediatric Cardiology and Adult Congenital Cardiology, Tokyo Women’s Medical University, 8-1 Kawada-cho, Shinjuku, Tokyo, 162-8666 Japan; 2 Department of Structural Heart Disease, The First Affiliated Hospital of Xi’an Jiaotong University, 277 Yanta West Road, Xi’an, 710061 Shaanxi China

**Keywords:** Pulmonary arterial hypertension, Silibinin, CXCR4, SDF-1, Stem cell, Smooth muscle cell, Lung

## Abstract

**Background:**

C-X-C chemokine receptor type 4 (CXCR4) may be involved in the development of pulmonary arterial hypertension (PAH). CXCR4 inhibitor AMD3100 was described to have a positive effect on the prevention of pulmonary arterial muscularization in PAH models. Silibinin is a traditional medicine that has an antagonistic effect on CXCR4. We investigated the effect of silibinin using rat models of PAH.

**Methods:**

PAH was induced by a single subcutaneous injection of monocrotaline. The rats were maintained in a chronic hypoxic condition (10% O_2_) with or without silibinin. To evaluate the efficacy of silibinin on PAH, right ventricular systolic pressure (RVSP), Fulton index (weight ratio of right ventricle to the left ventricle and septum), percent medial wall thickness (% MT), and vascular occlusion score (VOS) were measured and calculated. Immunohistochemical analysis was performed targeting CXCR4 and c-Kit. Reverse transcription-quantitative polymerase chain reaction was performed for the stem cell markers CXCR4, stromal cell derived factor-1 (SDF-1), c-Kit, and stem cell factor (SCF), and the inflammatory markers monocyte chemoattractant protein 1 (MCP1), interleukin-6 (IL-6), and tumor necrosis factor alpha (TNFα). Statistical analyses were performed using *t*-test and one-way analysis of variance with Bonferroni’s post hoc test.

**Results:**

Silibinin treatment for 1 week reduced RVSP and Fulton index. Treatment for 2 weeks reduced RVSP, Fulton index, % MT, and VOS, as well as downregulating the expression of CXCR4, SDF-1, and TNFα in pulmonary arteries. In contrast, treatment for 3 weeks failed to ameliorate PAH. The time-course study demonstrated that RVSP, Fulton index, % MT, and VOS gradually increased over time, with a decrease in the expression of CXCR4 and TNFα occurring after 2 weeks of PAH development. After 3 weeks, SDF-1, c-Kit, and SCF began to decrease and, after 5 weeks, MCP1 and IL-6 gradually accumulated.

**Conclusions:**

The CXCR4 inhibitor silibinin can ameliorate PAH, possibly through the suppression of the CXCR4/SDF-1 axis, until the point where PAH becomes a severe and irreversible condition. Silibinin results in reduced pulmonary arterial pressure and delays pulmonary arteriolar occlusion and pulmonary vascular remodeling.

**Electronic supplementary material:**

The online version of this article (10.1186/s12931-019-1041-y) contains supplementary material, which is available to authorized users.

## Background

The pathogenesis of pulmonary arterial hypertension (PAH) is intricate and involves endothelial dysfunction, chronic inflammation, smooth muscle cell proliferation, pulmonary arteriolar occlusion, apoptosis resistance, and pulmonary vascular remodeling. Right ventricular overload, right heart failure, and death may result from these severe pathologies [1–3]. No effective treatment has been discovered yet.

C-X-C chemokine receptor type 4 (CXCR4) plays a role in multiple pathways in carcinogenesis [4–6]. CXCR4 is expressed in stem/progenitor cells, including endothelial and smooth muscle progenitors [7]. The ligand of CXCR4 is stromal cell derived factor-1 (SDF-1), which is also termed C-X-C chemokine ligand 12 (CXCL12). Mesenchymal stem cells (MSCs) contribute to cancer progression through SDF-1/CXCR4 signaling [8]. However, cells in remodeled pulmonary arteries (PAs) of patients with pulmonary hypertension reportedly share similar characteristics with cancer cells, such as inflammation, increased proliferation, and decreased apoptosis [9]. Thus, we predict there is also an association between CXCR4/MSCs and the pathogenesis of PAH. CXCR4 and MSCs may also play a role in the chemotaxis, proliferation, and survival of smooth muscle cells, endothelial cells, or other cell types [10].

We previously reported that CXCR4 and other stem/progenitor cell markers, such as c-Kit, SCF, CD29, and CD90, are significantly higher in PAH rats than in normal rats [11]. Other studies have also reported that CXCR4 and/or c-Kit positive cells are critical in the development of pulmonary hypertension and vascular remodeling in rats [12–18]. While the small-molecule CXCR4 inhibitor AMD3100 (Plerixafor) prevents pulmonary arterial muscularization in several PAH models [12, 15–17], efficacious alternative CXCR4 inhibitors for PAH treatment are scarce.

We investigated the effect of another CXCR4 inhibitor. Silibinin is derived from the seeds of the milk thistle plant (*Silybum marianum* (L.)) [19]. Silibinin has been used in the clinical treatment of liver diseases [20] and it may be potentially useful to treat cancer [21–25]. Silibinin is a mixture of two flavonolignans, namely silybin A (Sil A) and sylybin B (Sil B), in a ratio approximately 1:1. Recently, using nematode Aβ amyloidogenesis model, it was discovered that Sil B completely inhibited amyloid β (Aβ) growth both in vitro and in vivo. On the other hand, Sil A did not show such an effect [26].

Silibinin and AMD3100 are thought to work in different ways [27–30]. We evaluated the ameliorative effect of silibinin over time using a rat monocrotaline (MCT) PAH model with chronic hypoxia exposure.

## Methods

### Animal preparation

MCT (Sigma-Aldrich, St. Louis, MO, USA) was dissolved in 1 N HCl, neutralized with 1 N NaOH, and diluted with distilled water to 20 mg/mL. A dose of 60 mg/kg (3 mL/kg) body weight was administered to the rats [31]. All rats had unlimited access to food and water and were weighed weekly. Silibinin (Sigma-Aldrich) was suspended in 0.5% carboxymethyl cellulose (CMC) sodium salt water (Wako Pure Chemical Industries, Ltd., Osaka, Japan).

Male, 7–8-week old Sprague-Dawley rats weighing 180–250 g (Tokyo Experimental Animal Company, Tokyo, Japan) were randomly assigned to eight groups. Four of the groups were PAH only groups. In these groups, the rats were subcutaneously injected with a single dose of MCT and maintained in a 10% hypoxic air chamber (Everest Summit II Altitude Generator) for 1, 2, 3, and 5 weeks. These groups were designated PAH-1w (*n* = 8), PAH-2w (*n* = 8), PAH-3w (*n* = 4) and PAH-5w (*n* = 8), respectively. Every rat in the PAH groups received CMC water without silibinin orally every day.

Three of the groups were PAH + silibinin. The rats were also subcutaneously injected with a single dose of MCT and maintained in a 10% hypoxic air chamber for 1 week (Sil-1w, *n* = 8), 2 weeks (Sil-2w, *n* = 8), and 3 weeks (Sil-3w, *n* = 4). Silibinin (200 mg/kg) with CMC water were supplied orally per day. An additional Sil-5w group is shown in Additional file 1.

Finally, in the control group (*n* = 8) the rats were subcutaneously injected with a single dose of 0.9% saline and maintained in a chamber with normal air for 5 weeks.

All animal experiment protocols were approved by the Institutional Animal Experiment Committee of the Tokyo Women’s Medical University (AE18–111). All animal procedures were in accordance with the ethical standards of the institution and conformed to the guidelines from Directive 2010/63/EU of the European Parliament on the protection of animals used for scientific purposes or the current NIH guidelines (NIH publication No. 85–23).

### Hemodynamic and functional studies

All rats were anesthetized by isoflurane inhalation (2.0% mixed with air, at an inhalation rate of approximately 350 mL/min) at each time point. Heart rate and respiratory monitoring were used to confirm the adequacy of anesthesia. A microtube was inserted into the right ventricle via the right jugular vein to measure the right ventricular systolic pressure (RVSP). The catheter was connected to a PowerLab Data Acquisition system and Lab Chart 7 software (ADInstruments, Dunedin, New Zealand) to record the data. The rats were sacrificed under isoflurane inhalation by cervical dislocation. Tissues (heart, lungs, and PAs) were isolated and harvested; the free wall of the right ventricle (RV) and the left ventricle and septum (LV + S) were separated and weighed to measure the Fulton index (weight ratio of RV to LV + S).

### Morphometric examinations

Immunohistochemical staining was performed as previously described [11]. Primary antibodies against alpha-smooth muscle actin (SMA, 1:1000; Sigma-Aldrich), proliferating cell nuclear antigen (PCNA, 1:500; Sigma-Aldrich), CXCR4 (1:1000; Abcam, Cambridge, UK), and c-Kit (1:100; Santa Cruz Biotechnology, Dallas, TX, USA) were used.

SMA staining was used to demonstrate smooth muscle cells in PA walls and to calculate the percent medial wall thickness (% MT). The external diameter (ED) and MT were measured in muscularized PAs, whose EDs varied from 50 to 100 μm, to calculate % MT = (2 × MT/ED) × 100 [32]. PCNA staining was used to calculate the vascular occlusion score (VOS) of PAs, which was categorized as Grade 0 (no evidence of neointimal formation), Grade 1 (< 50% luminal occlusion), or Grade 2 (> 50% luminal occlusion) [33]. For all evaluations, 15 intra-acinar PAs per section from each rat were randomly selected.

CXCR4 and c-Kit staining were performed to compare protein expression and the number of stem cells in lung tissue. Since CXCR4^+^ cells aggregated, it was impractical to count individual cells. Hence, only c-Kit^+^ cells were counted and then compared between or among the groups. For each type of staining, we randomly chose 15 microscopic areas per section from each rat.

### Reverse transcription-quantitative polymerase chain reaction (RT-qPCR)

RNA was isolated from the small PA or lung tissue in the left and right lower lobes of the lung using the RNeasy Mini Kit (QIAGEN, Hilden, Germany). Complementary DNA (cDNA) was synthesized using the PrimeScript™ RT Reagent Kit (Takara Bio, Shiga, Japan). qPCR was performed using a PikoReal Real-Time PCR System (Thermo Fisher Scientific, Waltham, MA, USA). Each sample was analyzed in triplicate. β-actin mRNA expression was measured for normalization. mRNA expression was normalized to β-actin expression using the 2^-∆∆CT^ equation. Primer sequences are listed in Additional file 1: Table S1.

### Statistical analyses

Quantitative data are expressed as mean ± standard deviation. Statistical analyses performed between two groups were analyzed by *t*-test. Comparison of the time-course was analyzed by one-way analysis of variance (ANOVA) and Bonferroni’s post hoc test. Comparison of the time course together with silibinin effect and other factors, including RVSP and VOS, was analyzed by two-way ANOVA. SPSS software (SPSS, Inc., Chicago, IL, USA) was used for all statistical analyses. A *p*-value < 0.05 was considered statistically significant.

## Results

### Animal survival rate

All rats in the control group and 1-, 2-, and 3-week groups survived and remained active during the experiment. In contrast, three rats in the PAH-5w group died after 4 weeks of the experimental treatment.

### Effect of silibinin with time in the PAH models

We used several doses of silibinin (0, 50, 100, 200 mg/kg), and found that it is effective in a dose-dependent manner (data not shown). Therefore, a dosage of 200 mg/kg silibinin was selected for subsequent experiments.

**Fig. 1:**
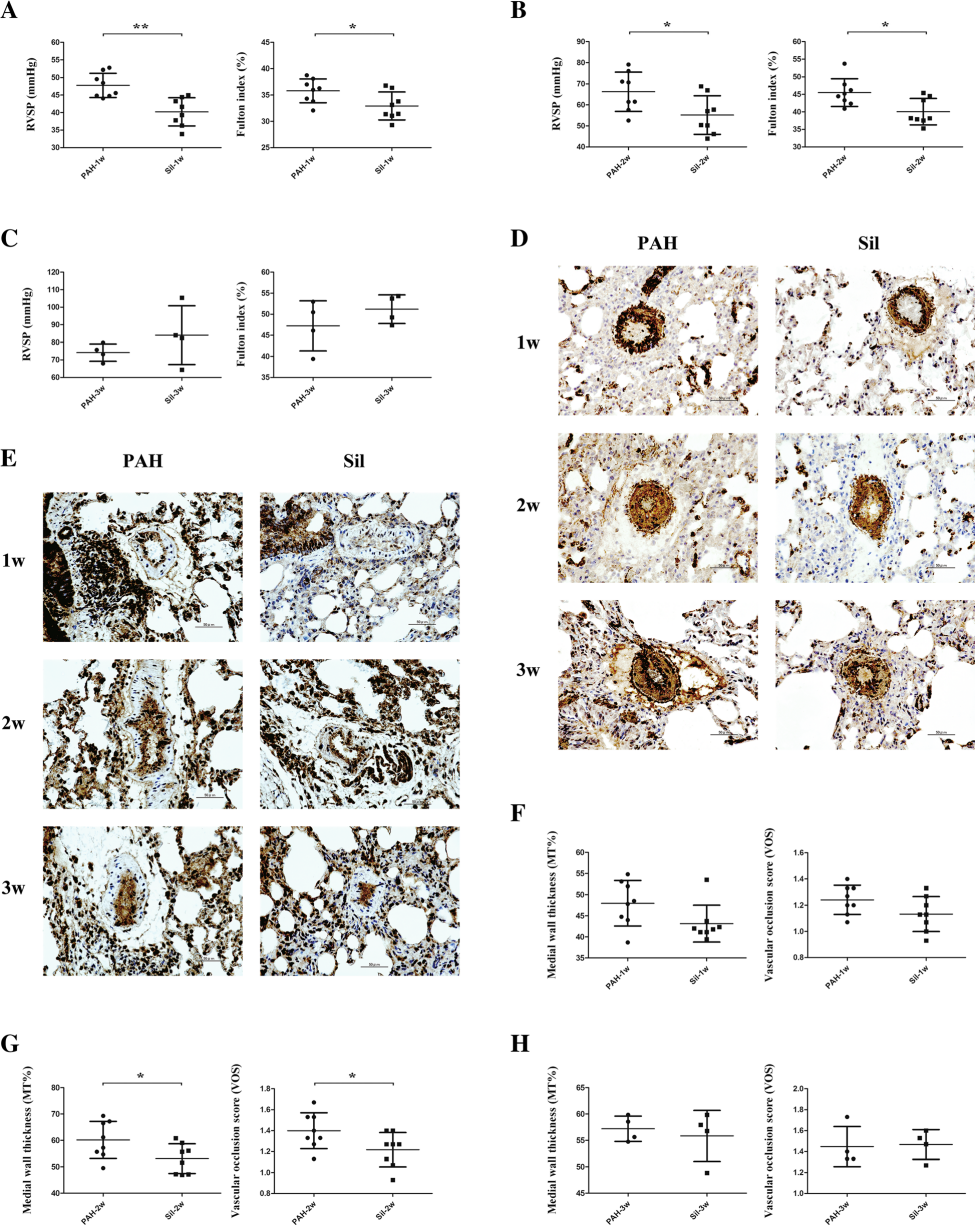
Hemodynamic studies and immunohistochemical evaluation of PAH models with and without silibinin. **a** Silibinin decreased RVSP and Fulton index in the Sil-1w group compared to the PAH-1w group. **b** Silibinin also decreased RVSP and Fulton index in the Sil-2w group compared to the PAH-2w. **c** Silibinin did not decrease RVSP and Fulton index during the 3-week experiment. **d** SMA staining revealed thinner media smooth muscle layer in the Sil-2w group compared to that in the PAH-2w group, but this was no longer found after 1 or 3 weeks. **e** PCNA staining revealed less proliferation in PAs in the Sil-2w group compared to PAH-2w, but no change was observed when compared to the 1- and 3-week treatment periods. **f**-**h** % MT and VOS were calculated at 1 week (**f**), 2 weeks (**g**), and 3 weeks (**h**) of treatment. Significant differences were found at the 2-week time point only (**g**); *p < 0.05 and **p < 0.01. Treatment groups consisted of eight rats each in the PAH-1w, PAH-2w, Sil-1w, and Sil-2w groups and four rats each in the PAH-3w and Sil-3w groups

**Fig. 2:**
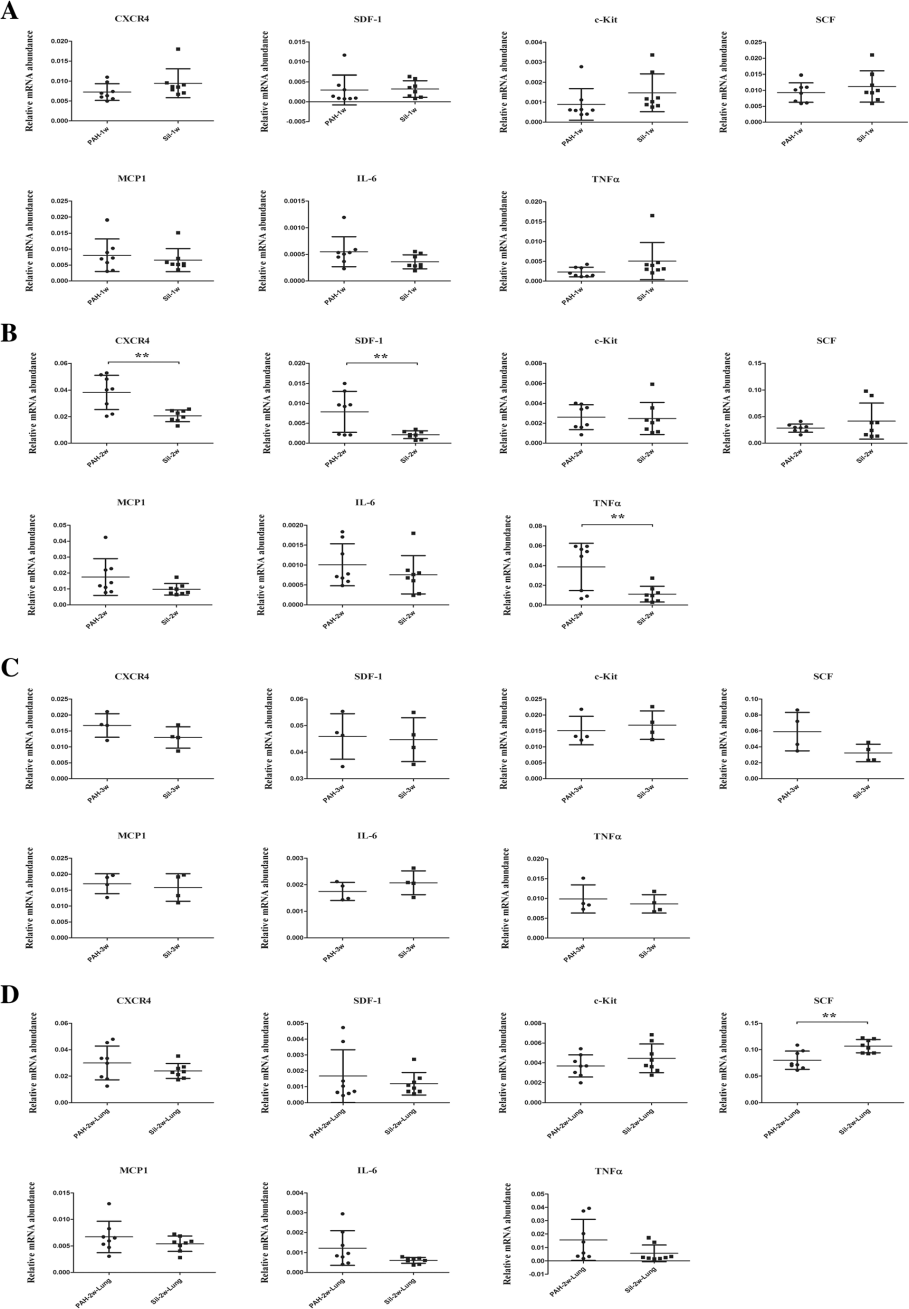
Gene expression levels of stem cell-related markers and inflammatory markers. Total RNA from PAs was used for reverse transcription (RT), and quantitative polymerase chain reaction (qPCR) was performed. The message was normalized to β-actin using the 2^-∆∆CT^ eq. **a** Silibinin did not downregulate the expression of stem cell-related and inflammatory markers in PAs after a 1-week treatment. **b** Silibinin significantly downregulated the expression of CXCR4, SDF-1 as well as TNFα in PAs after a 2-week treatment. **c** Silibinin did not downregulate the expression of stem cell-related and inflammatory markers in PAs after a 3-week treatment. **d** Silibinin did not downregulate the expression of stem cell-related and inflammatory markers in lung tissue after a 2-week treatment; **p < 0.01. Treatment groups consisted of eight rats each in the PAH-1w, PAH-2w, Sil-1w, and Sil-2w groups and four rats each in the PAH-3w and Sil-3w groups

Silibinin significantly decreased RVSP (*p* < 0.01) and the Fulton index (*p* < 0.05) after the 1-week treatment (Fig. 1a) and 2-week treatment (both *p* < 0.05, Fig. 1b). However, no significant difference was observed between the PAH-3w and Sil-3w groups in terms of hemodynamic levels (Fig. 1c). To further investigate the effectiveness of silibinin, staining was carried for SMA (Fig. 1d) and PCNA (Fig. 1e), and % MT as well as VOS were calculated (Fig. 1f-h). A significant difference was observed only for the 2-week treatment (*p* < 0.05, Fig. 1g).

RT-qPCR revealed that 1 week treatment of silibinin did not downregulate the expression of stem cell-related (CXCR4/SDF-1/c-Kit/SCF) and inflammatory (MCP1/IL-6/TNFα) markers in PAs (Fig. 2a). After 2 weeks, silibinin downregulated the expression of CXCR4, SDF-1, and TNFα in PAs, and a significant difference was observed between the PAH-2w and Sil-2w groups (*p* < 0.01). However, a significant difference was not observed in the expression of c-Kit, SCF, MCP1, and IL-6 in PAs between the PAH-2w and Sil-2w groups (Fig. 2b). After 3 weeks, silibinin failed to downregulate the expression of stem cell-related and inflammatory markers in PAs (Fig. 2c). To determine whether the strong expression of stem cells and inflammatory markers was specific to PAs, RT-qPCR was also done on lung tissue after 2 weeks of silibinin treatment. In contrast to PAs, silibinin did not downregulate the expression of stem cells and inflammatory markers in lung tissue (Fig. 2d).

**Fig. 3:**
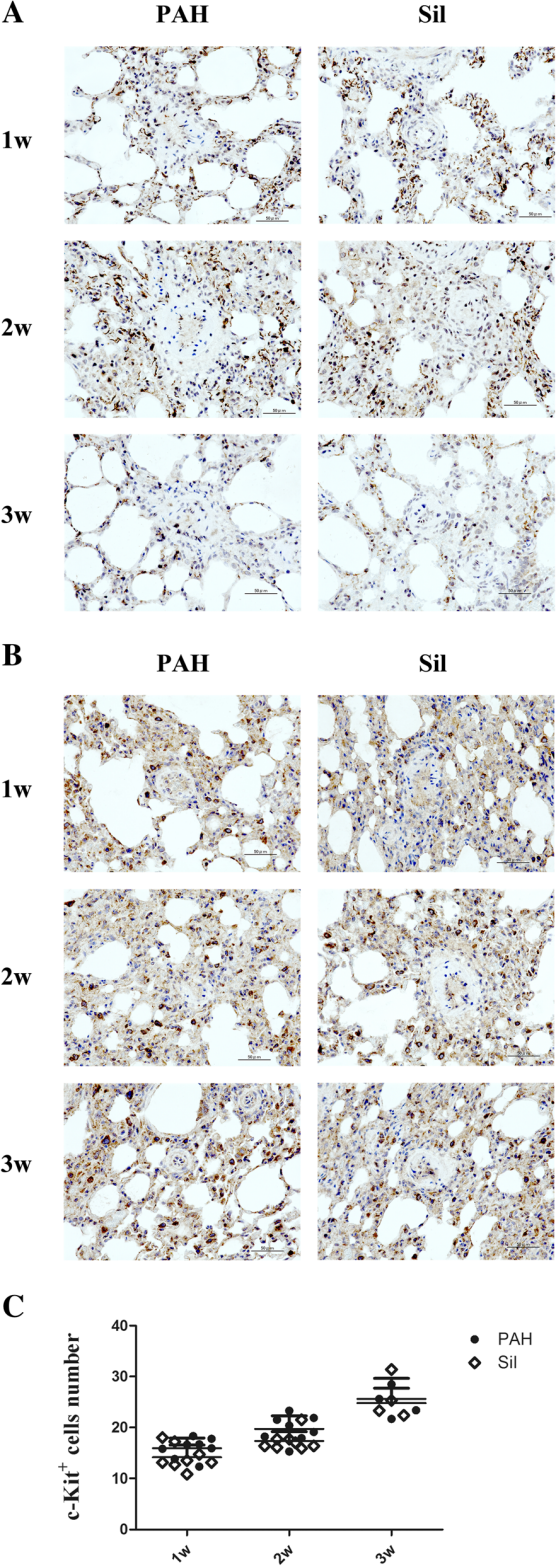
Immunohistochemical evaluation of CXCR4 and c-Kit around PAs. **a** More CXCR4^+^ cells after 2 weeks of treatment were observed compared to 1 and 3 weeks. **b** c-Kit^+^ cell numbers after the 3-week treatment was greater than those observed after the 1- and 2-week treatments. **c** There was no significant difference in c-Kit^+^ cell numbers between the PAH groups and silibinin groups at any time point. Treatment groups consisted of eight rats each in the PAH-1w, PAH-2w, Sil-1w, and Sil-2w groups and four rats each in the PAH-3w and Sil-3w groups

Immunohistochemical staining was performed to further investigate the efficacy of silibinin. CXCR4^+^ (Fig. 3a) and c-Kit^+^ cells (Fig. 3b) in close proximity to PAs were more strongly expressed than in other parts of the lung tissue. No significant difference in the number of c-Kit^+^ cells were observed at any time point (Fig. 3c).

### Time course results with PAH rats

**Fig. 4:**
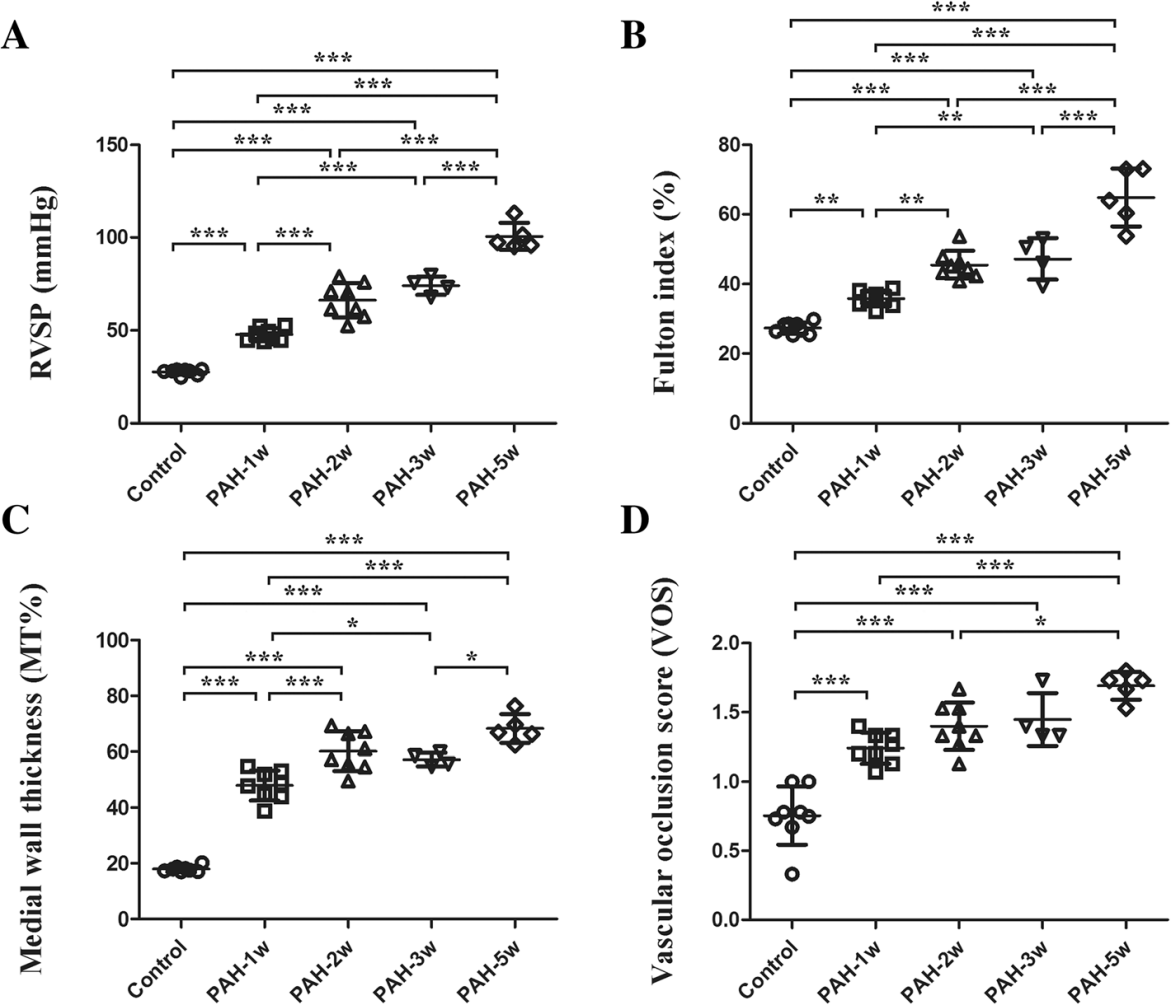
Time course of hemodynamic and immunohistochemistry of PAH models. **a**, **b** Significant differences were observed between each group, except between the PAH-2w and PAH-3w groups for RVSP (**a**) and Fulton index (**b**). **c** and **d** % MT (**c**) and VOS (**d**) increased over time within 5 weeks of PAH development; *p < 0.05, **p < 0.01, and ***p < 0.001. Treatment groups consisted of eight rats each in the control, PAH-1w, and PAH-2w groups, four rats in the PAH-3w group, and five rats in the PAH-5w group

We investigated stem cell and inflammatory marker expression during a time course study of the progression of PAH. Over time, gradual increases of RVSP (Fig. 4a), Fulton index (Fig. 4b), % MT (Fig. 4c), and VOS (Fig. 4d) were observed.

**Fig. 5:**
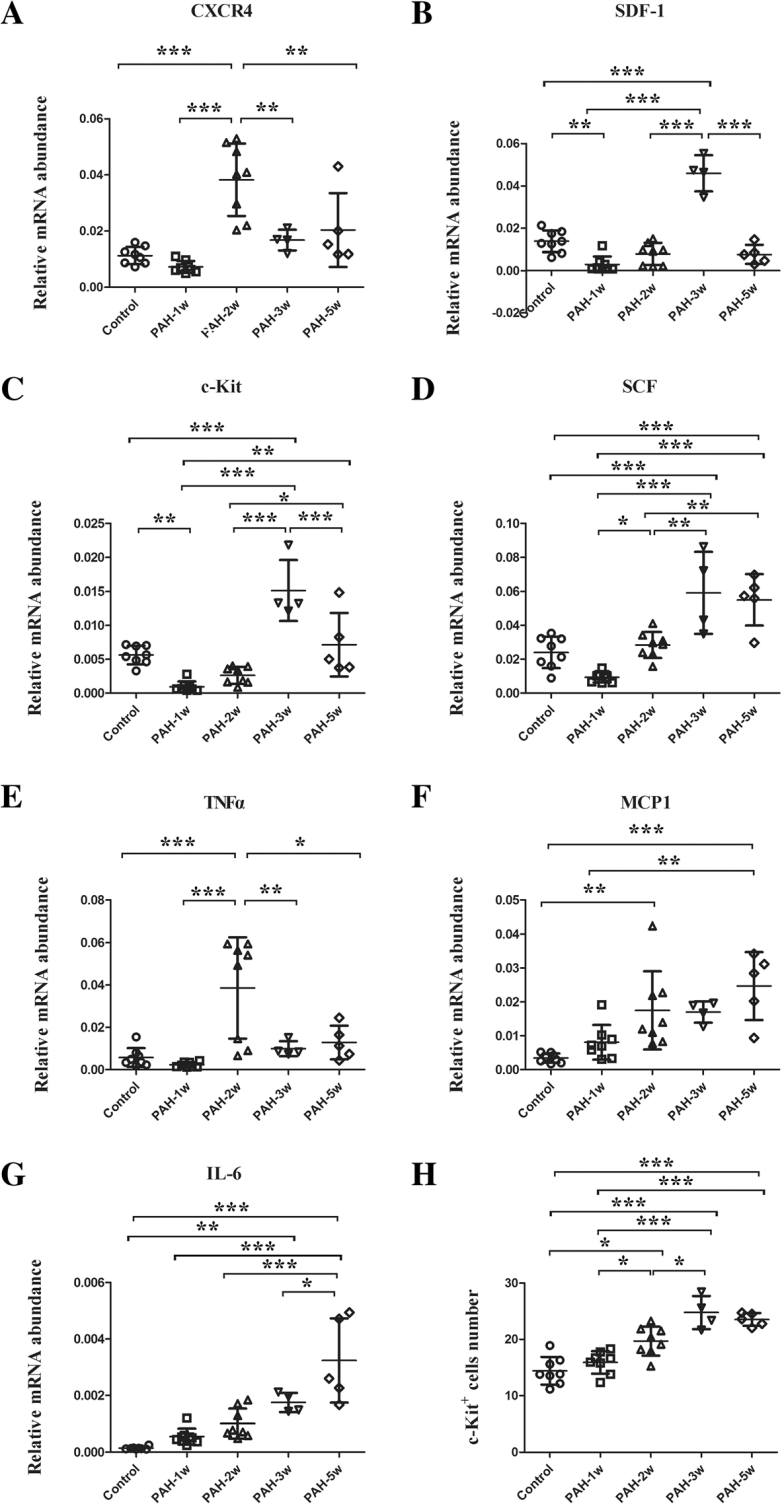
Expression of stem cell-related and inflammatory markers during the time course of PAH development. **a** CXCR4 gene expression in the PAH-2w group was significantly higher than in the control, PAH-1w, PAH-3w, and PAH-5w groups. After 2 weeks of PAH development, CXCR4 expression started to decrease. **b** SDF-1 gene expression level in the PAH-1w group was significantly less than the control group. After 1 week of PAH development, SDF-1 expression started to increase, and was significantly higher in the PAH-3w group than in the control, PAH-1w, PAH-2w, and PAH-5w groups. After 3 weeks of PAH development, SDF-1 expression started to decrease. **c** and **d** c-Kit (**c**) and SCF (**d**) gene expressions exhibited the same tendency and had results similar to SDF-1. **e** The change in the gene expression level of inflammatory marker, TNFα, was similar to that of CXCR4. **f** and **g** The gene expressions of inflammatory markers MCP1 (**f**) and IL-6 (**g**) gradually increased over time. **h** Immunohistochemical staining of c-Kit was compared in this time course study. c-Kit^+^ cell number in the PAH-2w group was significantly greater than in the control and PAH-1w groups. c-Kit^+^ cell number in the PAH-3w group was also significantly greater than in the control, PAH-1w, and PAH-2w groups. After 3 weeks of PAH development, c-Kit expression started to decrease; *p < 0.05, **p < 0.01, and ***p < 0.001. Treatment groups consisted of eight rats each in the control, PAH-1w, and PAH-2w groups, four rats in the PAH-3w group, and five rats in the PAH-5w group

Rats in the PAH-2w group exhibited significantly higher CXCR4 gene expression than the control (*p* < 0.001), PAH-1w (*p* < 0.001), PAH-3w (*p* < 0.01), and PAH-5w (*p* < 0.01) groups (Fig. 5a). Rats in the PAH-1w group exhibited significantly less SDF-1 mRNA expression levels than the control group (*p* < 0.01). After 1 week of the PAH condition, SDF-1 mRNA expression started to increase. SDF-1 gene expression was significantly higher in the PAH-3w group than in the control (*p* < 0.001), PAH-1w (*p* < 0.001), PAH-2w (*p* < 0.001), and PAH-5w (*p* < 0.001) groups (Fig. 5b). Results of the gene expression analysis of c-Kit and SCF were similar to those of SDF-1 (Fig. 5c, d). Additionally, gene expression of the inflammatory marker TNFα were similar to CXCR4 (Fig. 5e). Furthermore, a gradual increase in gene expression was found for two other inflammatory markers, MCP1 (Fig. 5f) and IL-6 (Fig. 5g). All stem cell markers examined showed decreased levels in the PAH-1w group compared to the normal group, but started to increase thereafter.

During the time course study, the number of c-Kit^+^ cells detected by immunohistochemical staining was greater in the PAH-2w group than in the control (*p* < 0.05) and PAH-1w groups (*p* < 0.05). Additionally, c-Kit^+^ cells in the PAH-3w group were significantly increased compared to the control (*p* < 0.001), PAH-1w (*p* < 0.001), and PAH-2w (*p* < 0.05) groups. However, their expression started to decrease thereafter (Fig. 5h). The results of two-way ANOVA are shown in Additional file 1.

## Discussion

There are four main findings of the present study. Firstly, a 1-week silibinin treatment in the PAH models reduced RVSP and Fulton index. A 2-week treatment reduced RVSP, Fulton index, % MT, VOS, and downregulated CXCR4, SDF-1 and TNFα gene expression in PAs. In contrast to these results, a 3-week treatment failed to ameliorate PAH symptoms. These results suggest that silibinin can ameliorate PAH and reduce pulmonary arterial pressure in the early phases of its development, but is an ineffective treatment in late stages of the disease. Furthermore, silibinin may delay pulmonary arteriolar occlusion and pulmonary vascular remodeling through the suppression of the CXCR4/SDF-1 axis. Secondly, after the 2-week silibinin treatment, stem cell-related and inflammatory markers were downregulated in PAs, but not in lung tissue. Thirdly, in the earliest period at which PAH development was measured (1-week MCT + hypoxia), the CXCR4 mRNA expression and other stem cell-related markers were lower than in normal rats. Fourthly, during this time course study, RVSP, Fulton index, % MT, and VOS continued to increase over time, but gene expression patterns were distinct, as CXCR4 and TNFα started to decline after 2 weeks of PAH. However, c-Kit, SCF, and SDF-1 were still accumulating after 2 weeks and started to decline after 3 weeks of PAH development. In contrast, the inflammatory markers MCP1 and IL-6 accumulated throughout the 5-week time period.

One week of silibinin treatment could ameliorate RVSP and Fulton index, however, no significant difference in gene expression was observed between the PAH-1w and Sil-1w groups. Simultaneously, in the time course study, we demonstrated that CXCR4 mRNA expression and other stem cell-related markers were lower than in normal rats. A plausible explanation for this finding is that at the onset of the severe stage, models need time to adapt. Thus, mRNA expression of most stem cells decreased compared with their normal healthy counterparts.

Two weeks of silibinin treatment could ameliorate PAH, as indicated by the downregulation of RVSP, Fulton index, % MT, VOS, and the CXCR4, SDF-1, and TNFα gene expression results. However, these differences were not observed in c-Kit, SCF, MCP1, and IL-6 gene expression levels, or c-Kit protein expression level. One possible reason is that silibinin downregulated the expression of CXCR4 and its SDF-1 ligand, and some of the inflammatory markers, but failed to have an effect on the other stem cell markers or inflammatory markers tested. This indicates silibinin may be different from another CXCR4 inhibitor, AMD3100.

Previously, AMD3100 treatment was shown to attenuate pulmonary angiogenesis by reducing the number of c-Kit^+^ cells in rat lungs [34], and reduce the number of proliferating c-Kit^+^ SMA^+^ cells in PAH rats [12]. CXCR4 was shown to autologously regulate its expression from the PTEN experiment [35], suggesting silibinin can function as a CXCR4 antagonist. CXCR4 has a large ligand binding cavity, and silibinin binds to transmembrane TM2 (Asp97), TM5 (Gln200), TM7 (Glu288), and EL2 (Cys186 and Asp187). However, AMD3100 binds to TM2 (Asp97), TM3 (His113, Tyr116, Thr117, and Leu120), TM4 (Asp171), TM5 (His203), TM6 (Tyr255), TM7 (Ser285 and Glu288) and EL2 (Asp187 and Arg188) [30]. Thus, we speculate that silibinin and AMD3100 are different CXCR4 inhibitors, with different structures and mechanisms of binding, leading to different efficacies in PAH treatment. In our experiments, silibinin may have ameliorated PAH only by suppressing the CXCR4/SDF-1 axis, independent of c-Kit. This contrasts with results previously found in AMD3100 experiments [12].

Silibinin treatment reduced pulmonary arterial pressure and pulmonary arteriolar occlusion, and slowed pulmonary vascular remodeling. Additionally, it is interesting that after a 2-week treatment, silibinin downregulated the gene expression of stem cell and inflammatory markers in PAs, but not in lung tissue. We also observed higher numbers of CXCR4^+^ and c-Kit^+^ cells around the PAs than in other parts of the lung tissue by immunohistochemical staining. From these results, we propose that the expression of CXCR4 and c-Kit may be greater in and around PAs instead of other parts of the lung tissue. Therefore, silibinin would be especially effective around PAs.

Three weeks of silibinin treatment could not ameliorate PAH at any level tested. Being housed under hypoxic conditions for a long period, PAH may have already become an irreversible condition. Therefore, the condition of the animals could not be ameliorated by CXCR4 inhibitors. Compared to rats in the PAH-3w group, rats in the PAH-5w group had poorer results in terms of RVSP, Fulton index, % MT, and VOS measurements because of the presence of more severe PAH. Intraperitoneal injection of silibinin did not produce a difference (Additional file 1).

In the time course study, we successfully established a PAH model using a combination of MCT and chronic hypoxia, which may be more similar to clinical PAH than a single treatment [36]. RVSP, Fulton index, % MT, and VOS gradually increased over time, in agreement with other PAH models that gradually become more severe.

CXCR4 is expressed in stem/progenitor cells, including endothelial and smooth muscle progenitors [7]. c-Kit is a common marker of progenitor and stem cells, including the lung stem cells, which can repopulate airways and vessels [37], and vascular endothelial stem cells, which can generate functional blood vessels [38]. SDF-1 is highly expressed in the bone marrow stromal cells, but it is also widely expressed throughout organs, such as the brain, heart, liver, and lung [39]. The results of our time course study indicated that even though PAH treatment became progressively more severe over time, gene expression changes were variable. For example, CXCR4 and TNFα started to decline after 2 weeks of the PAH condition, SDF-1, c-Kit, and SCF started to decline after 3 weeks, while MCP1 and IL-6 gradually accumulated over 5 weeks. One possible explanation is that CXCR4^+^ progenitor and stem cells differentiate into endothelial or smooth muscle cells at approximately 2 weeks in our PAH model. However, SDF-1^+^, c-Kit^+^, and SCF^+^ progenitor and stem cells differentiated into lung, vascular endothelial, or other cells around the third week of PAH development in our model. The inflammatory markers MCP1 and IL-6 gradually accumulated over time, which indicated more severe inflammation over time. However, in contrast to MCP1 and IL-6, TNFα expression was distinct. One possible reason is that at approximately 2 weeks, normal cell apoptosis peaked. TNFα expression reflected the apoptosis of these normal cells.

Although CXCR4^+^ and c-Kit^+^ cells were distributed in whole lung tissue, a greater number of positive cells were observed around the PAs than in other parts of the lung tissue, as previously described. We counted the number of c-Kit^+^ cells after 3 weeks of the PAH condition, and c-Kit expression started to decline. This suggested c-Kit expression correlates with its gene expression in PAs. In contrast, CXCR4^+^ cells aggregated, leading to difficulty counting individual cells. This was possibly because CXCR4 is widely expressed on endothelial cells, neutrophils, monocytes, hematopoietic, and tissue-committed stem cells [40], all of which may exist in lung tissue or PAs during PAH development.

The present study has some limitations. Firstly, rat models are less complex than clinical patients even though we established our model by combining MCT and chronic hypoxia. Secondly, we studied silibinin only, which may have a mechanism that is distinct from AMD3100 in PAH treatment. The role of other CXCR4 inhibitors to prevent PAH remains unclear. Thirdly, it is not clear which silibinin component, Sil A or Sil B, is effective for PAH. Silibinin is difficlut to dissolve in water. To improve its efficacy in vivo, chemically modified silibinin has been developed [41, 42]. The modified silibinin may be more effective than original sillibinin for PAH patients. Lastly, there is a possibility that higher dose of silibinin may be more efficacious. However, side effects of silibinin in PAH patients are still unknown. Therefore, further studies to compare efficacy and side effects of different CXCR4 inhibitors in PAH treatment are meaningful and necessary, and may provide more options for PAH clinical treatment.

## Conclusions

Our experimental results suggest that if the CXCR4 inhibitor silibinin is used to ameliorate PAH prior to PAH becoming a severe and irreversible condition, the CXCR4/SDF-1 axis may be suppressed, leading to a reduction in pulmonary arterial pressure and pulmonary arteriolar occlusion. Additionally, a deceleration of pulmonary vascular remodeling may occur. This study provided basic evidence supporting the use of the CXCR4 inhibitor silibinin for the treatment of PAH. Hence, silibinin may be another potential treatment for PAH and may provide a new option for some PAH patients.

## Additional file

Additional file 1: **Table S1.** Primers used for RT-qPCR. **Table S2.** The *p* value of two-way ANOVA analysis. **Figure S1.** Measurement of RVSP in different groups. **Figure S2.** The results of two-way ANOVA analysis**. Figure S3.** Hemodynamic studies, immunohistochemical evaluation, and gene expression of PAH-5w and Sil-5w groups. (ZIP 6575 kb)

## Abbreviations

ANOVA: One-way analysis of variance
CXCL12: C-X-C chemokine ligand 12
CXCR4: C-X-C chemokine receptor type 4
ED: External diameter
LV + S: Left ventricle and septum
MCT: Monocrotaline
MSCs: Mesenchymal stem cells
MT: Medial wall thickness
PAH: Pulmonary arterial hypertension
PAs: Pulmonary arteries
PCNA: Proliferating cell nuclear antigen
RV: Right ventricle
RVSP: Right ventricular systolic pressure
SDF-1: Stromal cell derived factor-1
SMA: α-smooth muscle actin
VOS: Vascular occlusion score

## References

[1] Tuder RM, Archer SL, Dorfmuller P, Erzurum SC, Guignabert C, Michelakis E, Rabinovitch M, Schermuly R, Stenmark KR, Morrell NW (2013). Relevant issues in the pathology and pathobiology of pulmonary hypertension. J Am Coll Cardiol.

[2] Ranchoux B, Antigny F, Rucker-Martin C, Hautefort A, Pechoux C, Bogaard HJ, Dorfmuller P, Remy S, Lecerf F, Plante S (2015). Endothelial-to-mesenchymal transition in pulmonary hypertension. Circulation..

[3] Galie N, Humbert M, Vachiery JL, Gibbs S, Lang I, Torbicki A, Simonneau G, Peacock A, Vonk Noordegraaf A, Beghetti M (2016). 2015 ESC/ERS guidelines for the diagnosis and treatment of pulmonary hypertension: the joint task force for the diagnosis and treatment of pulmonary hypertension of the European Society of Cardiology (ESC) and the European Respiratory Society (ERS): endorsed by: Association for European Paediatric and Congenital Cardiology (AEPC), International Society for Heart and Lung Transplantation (ISHLT). Eur Heart J.

[4] Furusato B, Mohamed A, Uhlen M, Rhim JS (2010). CXCR4 and cancer. Pathol Int.

[5] Wu Y, Zhang C, Xu W, Zhang J, Zheng Y, Lu Z, Liu D, Jiang K (2016). CXC motif chemokine receptor 4 gene polymorphism and cancer risk. Medicine (Baltimore).

[6] Teicher BA, Fricker SP (2010). CXCL12 (SDF-1)/CXCR4 pathway in cancer. Clin Cancer Res.

[7] Miller RJ, Banisadr G, Bhattacharyya BJ (2008). CXCR4 signaling in the regulation of stem cell migration and development. J Neuroimmunol.

[8] Torsvik A, Bjerkvig R (2013). Mesenchymal stem cell signaling in cancer progression. Cancer Treat Rev.

[9] McLaughlin VV, Archer SL, Badesch DB, Barst RJ, Farber HW, Lindner JR, Mathier MA, McGoon MD, Park MH, Rosenson RS (2009). ACCF/AHA 2009 expert consensus document on pulmonary hypertension a report of the American College of Cardiology Foundation Task Force on Expert Consensus Documents and the American Heart Association developed in collaboration with the American College of Chest Physicians; American Thoracic Society, Inc; and the Pulmonary Hypertension Association. J Am Coll Cardiol.

[10] Kawaguchi N, Zhang TT, Nakanishi T. Involvement of CXCR4 in Normal and abnormal development. Cells. 2019;8. 10.3390/cells8020185 PMC6406665 30791675

[11] Zhang T, Kawaguchi N, Hayama E, Furutani Y, Nakanishi T (2018). High expression of CXCR4 and stem cell markers in a monocrotaline and chronic hypoxia-induced rat model of pulmonary arterial hypertension. Exp Ther Med.

[12] Farkas D, Kraskauskas D, Drake JI, Alhussaini AA, Kraskauskiene V, Bogaard HJ, Cool CD, Voelkel NF, Farkas L (2014). CXCR4 inhibition ameliorates severe obliterative pulmonary hypertension and accumulation of C-kit(+) cells in rats. PLoS One.

[13] Favre S, Gambini E, Nigro P, Scopece A, Bianciardi P, Caretti A, Pompilio G, Corno AF, Vassalli G, von Segesser LK (2017). Sildenafil attenuates hypoxic pulmonary remodelling by inhibiting bone marrow progenitor cells. J Cell Mol Med.

[14] Huang X, Wu P, Huang F, Xu M, Chen M, Huang K, Li GP, Xu M, Yao D, Wang L (2017). Baicalin attenuates chronic hypoxia-induced pulmonary hypertension via adenosine A2A receptor-induced SDF-1/CXCR4/PI3K/AKT signaling. J Biomed Sci.

[15] Yu L, Hales CA (2011). Effect of chemokine receptor CXCR4 on hypoxia-induced pulmonary hypertension and vascular remodeling in rats. Respir Res.

[16] Drummond S, Ramachandran S, Torres E, Huang J, Hehre D, Suguihara C, Young KC (2015). CXCR4 blockade attenuates hyperoxia-induced lung injury in neonatal rats. Neonatology..

[17] Young KC, Torres E, Hatzistergos KE, Hehre D, Suguihara C, Hare JM (2009). Inhibition of the SDF-1/CXCR4 axis attenuates neonatal hypoxia-induced pulmonary hypertension. Circ Res.

[18] Gambaryan N, Perros F, Montani D, Cohen-Kaminsky S, Mazmanian M, Renaud JF, Simonneau G, Lombet A, Humbert M (2011). Targeting of c-kit+ haematopoietic progenitor cells prevents hypoxic pulmonary hypertension. Eur Respir J.

[19] Surai PF (2015). Silymarin as a natural antioxidant: an overview of the current evidence and perspectives. Antioxidants (Basel).

[20] Saller R, Meier R, Brignoli R (2001). The use of silymarin in the treatment of liver diseases. Drugs..

[21] Singh RP, Agarwal R (2006). Prostate cancer chemoprevention by silibinin: bench to bedside. Mol Carcinog.

[22] Kaur M, Agarwal R (2007). Silymarin and epithelial cancer chemoprevention: how close we are to bedside?. Toxicol Appl Pharmacol.

[23] Raina K, Agarwal R (2007). Combinatorial strategies for cancer eradication by silibinin and cytotoxic agents: efficacy and mechanisms. Acta Pharmacol Sin.

[24] Kroll DJ, Shaw HS, Oberlies NH (2007). Milk thistle nomenclature: why it matters in cancer research and pharmacokinetic studies. Integr Cancer Ther.

[25] Dhanalakshmi S, Singh RP, Agarwal C, Agarwal R (2002). Silibinin inhibits constitutive and TNFalpha-induced activation of NF-kappaB and sensitizes human prostate carcinoma DU145 cells to TNFalpha-induced apoptosis. Oncogene..

[26] Sciacca MFM, Romanucci V, Zarrelli A, Monaco I, Lolicato F, Spinella N, Galati C, Grasso G (2017). Inhibition of abeta amyloid growth and toxicity by silybins: the crucial role of stereochemistry. ACS Chem Neurosci.

[27] Scala S (2015). Molecular pathways. Targeting the CXCR4-CXCL12 axis--untapped potential in the tumor microenvironment. Clin Cancer Res.

[28] Singh RP, Agarwal R (2006). Mechanisms of action of novel agents for prostate cancer chemoprevention. Endocr Relat Cancer.

[29] Gazak R, Walterova D, Kren V (2007). Silybin and silymarin--new and emerging applications in medicine. Curr Med Chem.

[30] Wang Y, Liang WC, Pan WL, Law WK, Hu JS, Ip DT, Waye MM, Ng TB, Wan DC (2014). Silibinin, a novel chemokine receptor type 4 antagonist, inhibits chemokine ligand 12-induced migration in breast cancer cells. Phytomedicine..

[31] Morimatsu Y, Sakashita N, Komohara Y, Ohnishi K, Masuda H, Dahan D, Takeya M, Guibert C, Marthan R (2012). Development and characterization of an animal model of severe pulmonary arterial hypertension. J Vasc Res.

[32] Rondelet B, Kerbaul F, Motte S, van Beneden R, Remmelink M, Brimioulle S, McEntee K, Wauthy P, Salmon I, Ketelslegers JM, Naeije R (2003). Bosentan for the prevention of overcirculation-induced experimental pulmonary arterial hypertension. Circulation..

[33] Nishimura T, Vaszar LT, Faul JL, Zhao G, Berry GJ, Shi L, Qiu D, Benson G, Pearl RG, Kao PN (2003). Simvastatin rescues rats from fatal pulmonary hypertension by inducing apoptosis of neointimal smooth muscle cells. Circulation..

[34] Shen CC, Chen B, Gu JT, Ning JL, Zeng J, Yi B, Lu KZ (2018). AMD3100 treatment attenuates pulmonary angiogenesis by reducing the c-kit (+) cells and its pro-angiogenic activity in CBDL rat lungs. Biochim Biophys Acta.

[35] Nemenoff RA, Simpson PA, Furgeson SB, Kaplan-Albuquerque N, Crossno J, Garl PJ, Cooper J, Weiser-Evans MC (2008). Targeted deletion of PTEN in smooth muscle cells results in vascular remodeling and recruitment of progenitor cells through induction of stromal cell-derived factor-1alpha. Circ Res.

[36] Lan B, Hayama E, Kawaguchi N, Furutani Y, Nakanishi T (2015). Therapeutic efficacy of valproic acid in a combined monocrotaline and chronic hypoxia rat model of severe pulmonary hypertension. PLoS One.

[37] Kajstura J, Rota M, Hall SR, Hosoda T, D'Amario D, Sanada F, Zheng H, Ogorek B, Rondon-Clavo C, Ferreira-Martins J (2011). Evidence for human lung stem cells. N Engl J Med.

[38] Fang S, Wei J, Pentinmikko N, Leinonen H, Salven P (2012). Generation of functional blood vessels from a single c-kit+ adult vascular endothelial stem cell. PLoS Biol.

[39] Debnath B, Xu S, Grande F, Garofalo A, Neamati N (2013). Small molecule inhibitors of CXCR4. Theranostics..

[40] Ratajczak MZ, Majka M, Kucia M, Drukala J, Pietrzkowski Z, Peiper S, Janowska-Wieczorek A (2003). Expression of functional CXCR4 by muscle satellite cells and secretion of SDF-1 by muscle-derived fibroblasts is associated with the presence of both muscle progenitors in bone marrow and hematopoietic stem/progenitor cells in muscles. Stem Cells.

[41] Romanucci V, Agarwal C, Agarwal R, Pannecouque C, Iuliano M, De Tommaso G, Caruso T, Di Fabio G, Zarrelli A (2018). Silibinin phosphodiester glyco-conjugates: synthesis, redox behaviour and biological investigations. Bioorg Chem.

[42] Romanucci V, Gravante R, Cimafonte M, Marino CD, Mailhot G, Brigante M, Zarrelli A, Fabio GD. Phosphate-linked silibinin dimers (PLSd): new promising modified metabolites. Molecules. 2017;22. 10.3390/molecules22081323 PMC6152259 28800072

