# Supplementary figures and images for: Silibinin efficacy in a rat model of pulmonary arterial hypertension using monocrotaline and chronic hypoxia
Source: Respir Res. 2019 Apr 25;20:79. doi: 10.1186/s12931-019-1041-y (PMC6485095; doi:10.1186/s12931-019-1041-y)

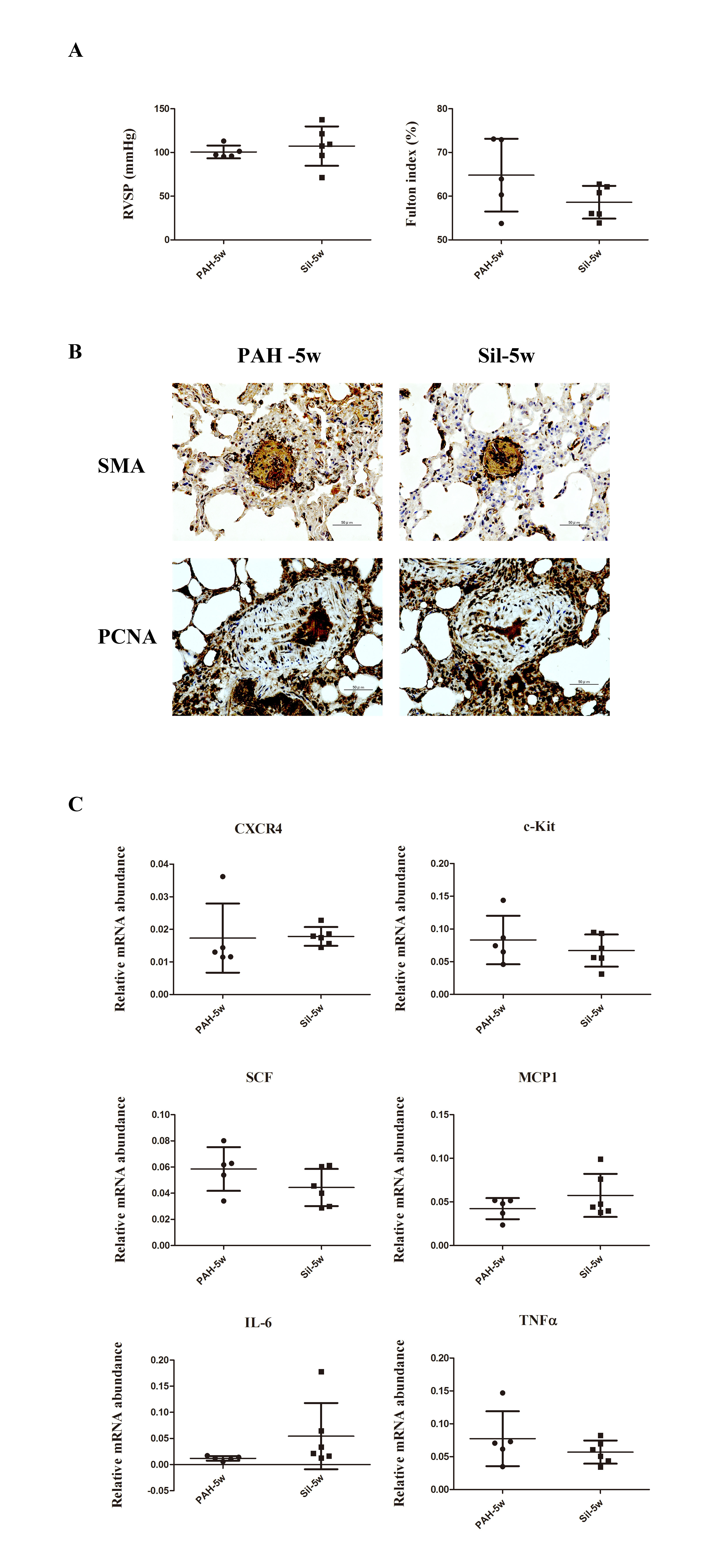

Supplement: Supplementary file 1 — Table S1. Primers used for RT-qPCR. Table S2. The p value of two-way ANOVA analysis. Figure S1. Measurement of RVSP in different groups. Figure S2. The results of two-way ANOVA analysis. Figure S3. Hemodynamic studies, immunohistochemical evaluation, and gene expression of PAH-5w and Sil-5w groups. (ZIP 6575 kb) [file 12931_2019_1041_MOESM1_ESM.zip › Sup_Fig3.jpg]

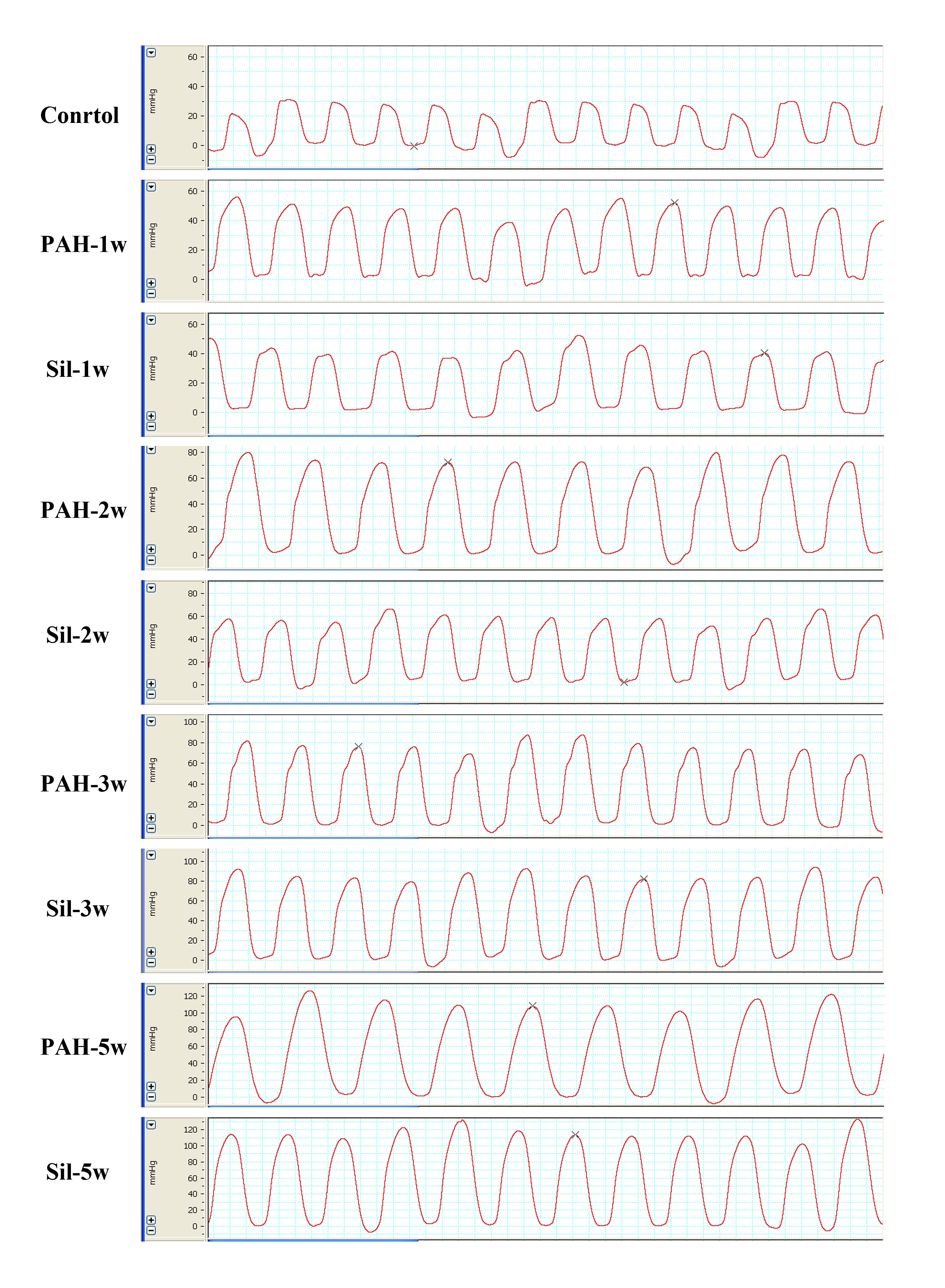

Supplement: Supplementary file 1 — Table S1. Primers used for RT-qPCR. Table S2. The p value of two-way ANOVA analysis. Figure S1. Measurement of RVSP in different groups. Figure S2. The results of two-way ANOVA analysis. Figure S3. Hemodynamic studies, immunohistochemical evaluation, and gene expression of PAH-5w and Sil-5w groups. (ZIP 6575 kb) [file 12931_2019_1041_MOESM1_ESM.zip › Sup_Fig1.jpg]
