# Supplementary material for: Silibinin efficacy in a rat model of pulmonary arterial hypertension using monocrotaline and chronic hypoxia
Source: Respir Res. 2019 Apr 25;20:79. doi: 10.1186/s12931-019-1041-y (PMC6485095; doi:10.1186/s12931-019-1041-y)
Supplement: Supplementary file 1 — Table S1. Primers used for RT-qPCR. Table S2. The p value of two-way ANOVA analysis. Figure S1. Measurement of RVSP in different groups. Figure S2. The results of two-way ANOVA analysis. Figure S3. Hemodynamic studies, immunohistochemical evaluation, and gene expression of PAH-5w and Sil-5w groups. (ZIP 6575 kb) [file 12931_2019_1041_MOESM1_ESM.zip › Table_S2.doc]

**Table S2** **The p value of two-way ANOVA analysis.**

|  | PAH-1w compare to Sil-1w | PAH-2w compare to Sil-2w | PAH-3w compare to Sil-3w | PAH-1w compare to PAH-2w | PAH-1w compare to PAH-3w | PAH-2w compare to PAH-3w | Sil-1w compare to Sil-2w | Sil-1w compare to Sil-3w | Sil-2w compare to Sil-3w |
| --- | --- | --- | --- | --- | --- | --- | --- | --- | --- |
| RVSP | 0.055 | 0.012* | 0.101 | 0.000*** | 0.000*** | 0.389 | 0.002** | 0.000*** | 0.000*** |
| Fulton index | 0.066 | 0.004** | 0.120 | 0.000*** | 0.000*** | 1.000 | 0.000*** | 0.000*** | 0.000*** |
| %MT | 0.017* | 0.008** | 0.708 | 0.000*** | 0.016* | 1.000 | 0.000*** | 0.000*** | 1.000 |
| VOS | 0.134 | 0.022* | 0.853 | 0.135 | 0.098 | 1.000 | 0.690 | 0.002** | 0.032* |
| CXCR4 | 0.519 | 0.000*** | 0.429 | 0.000*** | 0.079 | 0.000*** | 0.006** | 1.000 | 0.204 |
| SDF-1 | 0.841 | 0.019* | 0.716 | 0.127 | 0.000*** | 0.000*** | 1.000 | 0.000*** | 0.000*** |
| c-Kit | 0.636 | 0.906 | 0.277 | 0.392 | 0.000*** | 0.000*** | 1.000 | 0.000*** | 0.000*** |
| SCF | 0.860 | 0.151 | 0.042* | 0.119 | 0.000*** | 0.025* | 0.005** | 0.174 | 1.000 |
| MCP1 | 0.630 | 0.021* | 0.796 | 0.017* | 0.086 | 1.000 | 0.932 | 0.069 | 0.397 |
| IL-6 | 0.395 | 0.209 | 0.246 | 0.077 | 0.000*** | 0.012* | 0.193 | 0.000*** | 0.000*** |
| TNFα | 0.643 | 0.000*** | 0.882 | 0.000*** | 0.895 | 0.001** | 0.947 | 1.000 | 1.000 |

*p < 0.05, **p < 0.01, ***p < 0.001.
