# Supplementary material for: Silibinin efficacy in a rat model of pulmonary arterial hypertension using monocrotaline and chronic hypoxia
Source: Respir Res. 2019 Apr 25;20:79. doi: 10.1186/s12931-019-1041-y (PMC6485095; doi:10.1186/s12931-019-1041-y)
Supplement: Supplementary file 1 — Table S1. Primers used for RT-qPCR. Table S2. The p value of two-way ANOVA analysis. Figure S1. Measurement of RVSP in different groups. Figure S2. The results of two-way ANOVA analysis. Figure S3. Hemodynamic studies, immunohistochemical evaluation, and gene expression of PAH-5w and Sil-5w groups. (ZIP 6575 kb) [file 12931_2019_1041_MOESM1_ESM.zip › Table_S1_032918.doc]

**Table S1** Primers used for RT-qPCR

| Gene | Forward primer | Reverse primer |
| --- | --- | --- |
| CXCR4 | 5'-GCTGAGGAGCATGACAGACA-3' | 5'-GATGAAGGCCAGGATGAGAA-3' |
| SDF-1 | 5'-TTTGGTCAGAGGAAGAGGGG-3' | 5'-GTTTCACTACGGGCCACATC-3' |
| c-Kit | 5'-GATCTGCTCTGCGTCCTGTT-3' | 5'-AGATGGCTGAGAAGTCCCTGT-3' |
| SCF | 5'-TCGTGGCATGTATGGAAGAA-3' | 5'-TCAGATGCCACCATGAAGTC-3' |
| MCP1 | 5'-AGCATCCACGTGCTGTCTC-3' | 5'-GATCATCTTGCCAGTGAATGAG-3' |
| IL-6 | 5'-CCGGAGAGGAGACTTCACAG-3' | 5'-ACAGTGCATCATCGCTGTTC-3' |
| TNFα | 5'-TGACCCCCATTACTCTGACC-3' | 5'-GGCCACTACTTCAGCGTCTC-3' |
| ß-actin | 5'-CTAAGGCCAACCGTGAAAAG-3' | 5'-GCCTGGATGGCTACGTACA-3' |

CXCR4: C-X-C chemokine receptor type 4

SDF-1: stromal cell derived factor-1

SCF: stem cell factor

MCP1: monocyte chemoattractant protein 1

IL-6: interleukin-6

TNFα: tumor necrosis factor alpha
